# Economic evaluations of health care interventions in oropharyngeal dysphagia after stroke: protocol for a systematic review

**DOI:** 10.1186/s13643-022-01969-6

**Published:** 2022-05-14

**Authors:** Sergio Marin, Mateu Serra-Prat, Omar Ortega, Pere Clavé

**Affiliations:** 1 Gastrointestinal Physiology Laboratory, Hospital de Mataró, Universitat Autònoma de Barcelona, Mataró, Catalunya Spain; 2 Pharmacy Department, Hospital Universitari Germans Trias i Pujol, Badalona, Catalunya Spain; 3 Centro de Investigación Biomédica en Red de enfermedades hepáticas y digestivas (CIBERehd), Instituto de Salud Carlos III, Barcelona, Catalunya Spain; 4 Research Unit, Consorci Sanitari del Maresme, Mataró, Catalunya Spain

**Keywords:** Stroke, Brain ischemia/complications, Cerebral hemorrhage, Deglutition disorders, Deglutition, Economics, Health resources

## Abstract

**Background and purpose:**

Oropharyngeal dysphagia (OD) affects 40–81% of patients after stroke. A recent systematic review on the costs of OD and its main complications showed higher acute and long-term costs for those patients who developed OD, malnutrition and pneumonia after stroke. These results suggest that appropriate management of post-stroke OD could reduce clinical complications and costs. The purpose of this systematic review is to assess the available literature for healthcare interventions that are efficient or cost-effective in the management of OD.

**Methods:**

A systematic review on economic evaluations of health care interventions will be performed on post-stroke patients with OD following PRISMA recommendations. Four bibliographic databases will be searched and a subsequent reference check will be done. English and Spanish literature will be included without date restrictions. Studies will be included if they refer to economic evaluations or in which cost savings were reported in post-stroke patients suffering OD. Studies will be excluded if they are partial economic evaluation studies, if they refer to esophageal dysphagia, or if OD is caused by causes different from stroke. Evidence will be presented and synthetised with a narrative method and using tables. Quality evaluation will be done using the Consolidated Health Economic Evaluation Reporting Standards (CHEERS) statement.

**Discussion:**

The protocol for this systematic review is the first step to assess the cost-effectiveness of the healthcare interventions that have been described as potential treatments for post-stroke OD. This systematic review will summarise the current evidence on the relation between cost and benefits associated with the appropriate management of OD in post-stroke patients.

**Trial registration:**

PROSPERO CRD42020136245

## Introduction

Oropharyngeal dysphagia (OD) is a common complication in patients who have suffered from stroke; its prevalence in this population varies from 40 to 81% [1]. Post-stroke OD causes severe complications such as dehydration, malnutrition, respiratory infections including aspiration pneumonia, and hospital readmissions [1–3]. We previously found OD was an independent risk factor for prolonged hospital stay and institutionalisation after discharge, poorer functional capacity and increased mortality [2]. In addition, OD after stroke was associated with higher costs than that of stroke patients without OD [4, 5]. We also found OD caused significantly higher economic costs during hospitalisation that strongly and significantly increased with the development of malnutrition and respiratory infections at long-term follow-up. Patients with OD incurred higher total hospitalisation costs (€5,357.67 ± €3,391.62 vs. €3,976.30 ± €1,992.58, *p* < 0.0001), 3-month costs (€8,242.0 ± €5,376.0 vs. €5,320.0 ± €4,053.0, *p* < 0.0001), and 12-month costs (€11,617.58 ± €12,033.58 vs. €7,242.78 ± €7,402.55, *p* < 0.0001). Patients with OD who were at risk of malnutrition or malnourished and suffered at least one episode of respiratory infection incurred higher mean costs at 12-months’ follow-up compared with patients who did not developed OD (€19,817.58 ±€13,724.83 vs. €7242.8 ± €7402.6, *p* < 0.0004) [6].

Appropriate management and treatment of post-stroke OD is required to avoid clinical complications, improve patient quality of life and save healthcare resources [3]. Current treatment for post-stroke OD is not standardised and includes a series of strategies that can go from compensation measures, including fluid thickening and texture-modified foods (classical treatment) [7], to more innovative neurorehabilitation approaches including peripheral stimulation treatments (electrical and pharmacologic studies) and non-invasive brain stimulation techniques (NIBS) [8]. Compensatory strategies are the most used ones and can range from adaptation of fluids and diets with thickeners and texture-modified food respectively to the use of postures and manoeuvers compensating biomechanical deficits during deglutition [9]; however, these strategies do not improve swallowing physiology [8]. Neurorehabilitation strategies are based on the recovery of swallowing function and include peripheral stimulation treatments (transcutaneous and intrapharyngeal electrical stimulation and pharmacological transient receptors potential channels stimulants) and central stimulation treatments such as transcranial direct current stimulation (tDCS) or repetitive transcranial magnetic stimulation (rTMS) [8, 10, 11].

Although the application of an appropriate treatment for OD in post-stroke patients should be mandatory in the neurology wards and also during the follow up due to the high clinical, psychological and economic impact of OD [2, 5, 12, 13], currently, the cost-effectiveness of all the mentioned strategies is not well known. Thus, the aim of this study is to develop a protocol for a systematic review to assess and summarise the current evidence on the efficiency or cost-effectivenes of available healthcare interventions, including compensatory and innovative neurorehabilitation strategies, on the appropriate management of OD.

## Methods

### Protocol and registration

A systematic review will be performed on the economic evaluations of healthcare interventions on OD in patients who suffered a stroke. This systematic review will follow the recommendations stated by the Preferred Reporting Items for Systematic Reviews and Meta-Analysis (PRISMA) [14]. This protocol for a systematic review has been reported following the recommendations stated by PRISMA protocols annex (PRISMA-P) [15]. The protocol for this systematic review was registered in the International Prospective Register of Systematic Reviews of the Center for Reviews and Dissemination (PROSPERO) (registration number: CRD42020136245) [16]. The main outcome of interest will be the costs and the associated health benefits of available sanitary/healthcare interventions on post-stroke OD.

### Literature search

We will search MEDLINE using PubMed, Embase using Ovid, the National Health Service Economic Evaluation Database using the Center for Reviews and Dissemination Database of the University of York and the Cost-Effectiveness Analysis Registry database of the Center for the Evaluation of Value and Risk in Health. These databases will be searched up to 31th December 2020. A publication date restriction will not be imposed and English and Spanish literature will be included. After searching, EndNote software (EndNote 20, Clarivate, 2022) will be used to organise articles. This systematic review will not include posters, abstracts, book chapters or unpublished literature. Search strategy (combined MeSH and search terms used) applied at PubMed is described in Table 1. A similar strategy will be used in the other databases.

**Table 1:** Search terms and MeSH terms used in the bibliographic search

Terms related to oropharyngeal dysphagia and connected among themselves by “OR”	Terms related to stroke and connected among themselves by “OR”	Terms related to economic evaluations and connected among themselves by “OR”
1. ”Deglutition”[Mesh] 2. “Deglutition Disorders"[Mesh] 3. "Oropharynx/abnormalities"[Mesh] 4."Oropharynx/diagnosis"[Mesh] 5."Oropharynx/diagnostic imaging"[Mesh] 6."Oropharynx/pathology"[Mesh] 7."Oropharynx/pharmacology"[Mesh] 8."Oropharynx/physiopathology"[Mesh] 9."Oropharynx/therapy"[Mesh] 10.Enteral tube feed*/ 11.Swallow[ti/abs] 12.Dysphag*[ti/abs] 13.Deglut*[ti/abs] 14.Dysphagia[tw] 15.Dysphag*/ 16.Dysphagia therapy/	17.”Stroke”[Mesh] 18."Stroke Rehabilitation" [Mesh] 19. "Brain Ischemia/ complications"[Mesh] 20. “Cerebral Infarction"[Mesh] 21.” Cerebral Hemorrhage"[Mesh] 22.”Intracranial Embolism and Thrombosis”[Mesh] 23. “Intracranial Hemorrhages”[Mesh] 24.”Intracranial Arteriosclerosis”[Mesh] 25.”Cerebrovascular Disorders”[Mesh] 26.Stroke[ti/abs] 27.Post stroke[ti/abs] 28.Poststroke[ti/abs] 29.Post-stroke[ti/abs] 30.Cerebral Ischaemia[ti/abs] 31.Brain Ischaemia[ti/abs] 32.Brain infarct[ti/abs] 33.Intracranial hemorrhage[ti/abs] 34.Intracranial haemorrhage[ti/abs] 35.Cerebral Hemorrhage[ti/abs] 36.Cerebral Haemorrhage[ti/abs] 37.Brain Hemorrhage[ti/abs] 38.Brain Haemorrhage[ti/abs] 39.Stroke discharge/ 40.Post-stroke/	41.”Economics”[Mesh] 42."Economics" [Subheading] 43."Models, Economic"[Mesh] 44. Health Resources"[Mesh] 45. “Tertiary Care Centers/economics"[Mesh] 46. "Rehabilitation Centers/economics"[Mesh] 47."Length of Stay/ economics"[Mesh] 48. "Medicare/economics" [Mesh] 49. “Physical Therapy Modalities/economics"[Mesh] 50. “Emergency Medical Services/economics" [Mesh] 51. "Food, Formulated/ economics"[Mesh] 52. “Cerebrovascular Disorders/ economics"[Mesh] 53.Cost effectiveness analysis 54.Cost utility analysis 55.Cost minimization analysis 56.Cost benefit analysis 57.Cost[tw] 58.Costs[tw] 59.Quality-adjusted life years/ 60.Cost utility[ti/abs] 61.Cost-utility[ti/abs] 62.Cost benefit[ti/abs] 63. Cost-benefit[ti/abs] 64.Cost minimization[ti/abs] 65-Cost-minimization[ti/abs] 66.Cost effectiveness[ti/abs] 67.Cost-effectiveness[ti/abs]

Terms, detailed in the three columns above, related to oropharyngeal dysphagia, stroke and health economics will be connected using “AND”

### Selection process including eligibility criteria

**Fig. 1:**
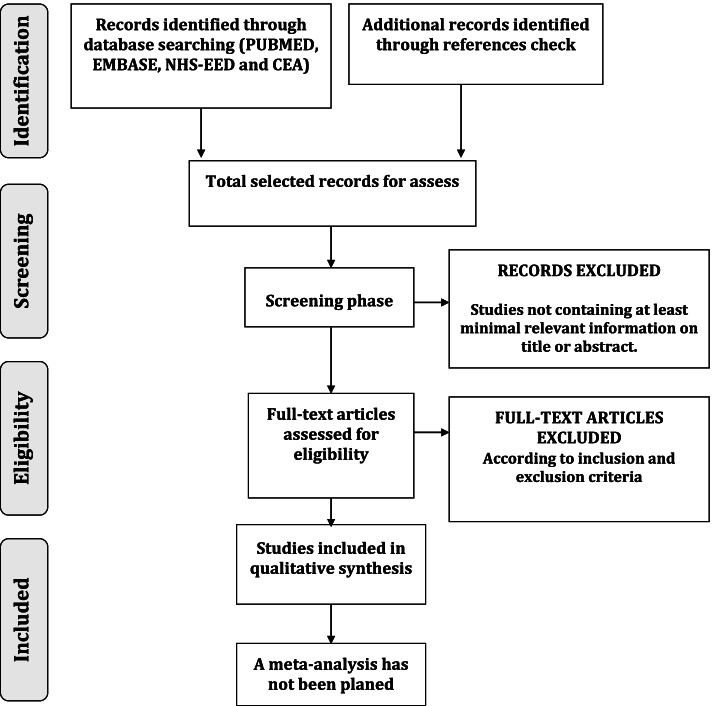
Selection process, flow diagram

Studies will be identified through literature search and will be selected using a double-phase process. In the first phase, one reviewer (SM) will assess the title and abstract of the identified articles. These articles will be excluded if they do not contain at least minimal relevant information about: “stroke” or “cerebral infarction” or “cerebral hemorrhage” or “brain ischemia”, “dysphagia” or “deglutition” or “swallowing assessment” or “swallowing disorders” and, “economic evaluation” or “economics” or “economic models” or “costs” in their abstracts or titles. A second reviewer (OO) will check the excluded articles using the same criteria. In the second phase, articles will be selected according to eligibility criteria. We will include articles if they have economic evaluations of the effect of the intervention on healthcare (cost minimisation studies, cost-utility studies, cost-effectiveness studies, cost-benefit analysis) or studies in which costs saving applying interventions in OD management are assessed (for instance, studies in which there are potential savings due to post-stroke patients being given different interventions to the usual management) and provide information on post-stroke adult patients (≥ 18 years) with OD. Studies will be excluded if they have only partial economic evaluation studies (cost of illness studies, cost-description studies, costs analysis or cost-consequence analysis among others), if they refer to esophageal dysphagia, OD caused by causes other than stroke or if there are duplicate publications from the same study (in that case only one will be considered). Two independent reviewers will perform this process independently (SM, OO). Subsequently, the results will be compared and disagreement between reviewers will be assessed using the Cohen’s kappa coefficient [17]. Finally, a third reviewer (MS-P or PC) will decide over conflicting results (Fig. 1).

### Data collection

One reviewer (SM) will extract all data from included studies in a specific data sheet (Microsoft Excel Software, USA, 2022). Total articles will be divided in three groups and each of the other three reviewers (OO, MS-P, PC) will extract data from one group using the same datasheet. The extracted data will be compared and, in case of disagreement, a third reviewer will participate in order to reach a final decision (OO, MS-P, PC). We will gather data related to the design, the participants, the quality and the results of each study. All study data will be transferred from the data collection form to a specific datasheet for each study and will include (a) study identification: first author, title, journal and year of publication; (b) Main study characteristics: aim, type of economic evaluation (cost minimisation, cost-utility, cost-effectiveness, cost-benefit, cost savings), intervention/s assessed, eligibility criteria, retrospective or prospective data gathering, data source, time horizon, economic perspective (patient, hospital, payer, healthcare system or society), country, year, currency, use of a temporary discount rate (yes/no), presence of a sensitivity analysis (yes/no), data source, location/setting; (c) study sample characteristics (if applicable, studies could use an economic model of the disease): sample size, sociodemographic data (age, average and range; gender), stroke type, mean value of National Institutes of Health Stroke Scale (NIHSS) or Canadian Neurologic Scale (if available on admission or discharge or during hospitalisation); (d) Description of the healthcare intervention assessed; (e) Elements of cost considered, all of them (yes/no): direct healthcare costs (hospitalisation: emergency room, hospitalisation ward, intensive care unit; long-term care: nursing home, social and health center, hospitalisation at home; primary care, medication, ambulance, special diets, tube-feeding, percutaneous endoscopic gastrostomy (PEG) insertion, outpatient visits: nutritionist, physical therapist, speech therapist, nurse; complication related costs: pneumonia, malnutrition), direct non-healthcare costs (social services, time, transportation) and, indirect costs (loss of productivity or time off from work, morbidity, mortality and/or impairment); (f) Specific data depending on the type of economic evaluation: for cost minimisation studies, the data related to the equivalence of interventions; for cost-utility studies, the data on the quality-adjusted life years (QUALYs) and/or the disability-adjusted life years (DALYs) applied; for cost-effectiveness studies, the effect units applied and for cost-benefit studies, the measured benefits; and (g) Results of studies depending on the type of economic evaluation: for cost minimisation studies, the economic savings by applying the most efficient intervention; for cost-utility studies, the incremental cost-utility ratio (ICUR); for cost-effectiveness studies, the incremental cost-effectiveness ratio (ICER) and for cost-benefit studies, the cost-benefit ratio. These data will be reported in their original format. If necessary, we will contact study authors of eligible articles to answer questions about unreported information or to clarify possible misunderstandings. Data obtained from study authors that is not available in the original articles will be clearly identified. We will not plan any calculation based on study data nor any assumption resulting from lost or unavailable information. Moreover, any assumption resulting from lost or unavailable information will be reported.

### Quality assessment

A specific tool to assess the internal validity and the reporting key factors of economic evaluation studies will be used. We will apply the Consolidated Health Economic Evaluation Reporting Standards (CHEERS) statement 2022 updated version [18]. A set of items that apply to a critical appraisal of economic evaluation studies is provided in this checklist. Each item represents a study aspect that we will rate as “yes, partly, no or not applicable”. For each study, the total amount of items will be rated as yes (1 point) and partly (0.5 points) and then it will be divided between the total applicable items. This total score will be expressed as a percentage; a higher score will represent a lower risk of bias. We will consider a score of 100% as a very low risk of bias study. As we want to assess the current state of the literature on this topic and not to create a final sum of the evidence, we will not exclude any study based on its quality assessment score from this review.

### Data presentation and data synthesis

We will use two different strategies to synthesise the information of this systematic review. A narrative method will be used to describe main characteristics of the study and the study sample, cost elements considered and specific data depending on the type of economic evaluation. Identification of studies, results and global score on quality assessment will be presented in a table. Finally, a meta-narrative synthesis of the extracted information will be performed where we will describe the evidence on the efficiency/cost effectiveness of different clinical interventions for post-stroke OD together with some of the key aspects of quality assessment evaluation. We will present results in the following order: (1) studies reporting data on interventions related to the screening or assessment of OD, (2) studies reporting data on compensatory treatment strategies, (3) studies reporting data on nutritional support by enteral tube feeding on patients with OD, (4) studies reporting data on rehabilitation programs that comprise interventions on OD management and (5) studies reporting data on restorative strategies. If a study reports on more than one of these interventions, data will be presented separately. Whenever available, data comparing two or more of these interventions (e.g. compensatory vs. restorative strategies) will be considered. Information on the study setting, economic perspective, and country will be considered and used to order and compare study results according to their relevance on the efficiency/cost effectiveness of the assessed interventions. Moreover, we will report it when studies identify, measure and assess all the important costs for each assessed alternative, whether the study structure (study approach, data and cost source) is performed in the most appropriate way to answer the study question and whether the most important factors to understand the economic evaluation are properly reported. Information will be presented according to the Centre for Reviews and Dissemination recommendations [19]. Finally, the possibility of creating an economic model of the disease will be studied and we will evaluate which pharmacoeconomic studies need to be carried out in order to understand the cost-effectiveness of post-stroke OD management.

## Discussion

The appropriate management of OD in post-stroke patients is imperative during acute stroke hospitalisation and during patient rehabilitation beyond acute care due to the potential clinical severity of its complications and also due to the high health-economic costs of these events. We previously found prevalence of post-stroke OD during admission in a general hospital was 45% and remained high at 3-months follow-up. OD after stroke was an independent risk factor for prolonged hospital stay (*P* = .049; β = 0.938) and institutionalisation after discharge (OR = 0.47; CI = 0.24–0.92); OD was an independent risk factor for poorer functional capacity (OR = 3.00; CI = 1.58–5.68) and increased mortality (HR = 6.90; CI = 1.57–30.34) 3 months after stroke [2]. Post-stroke OD is a dynamic condition and, although some spontaneous recoveries can be observed in patients with an optimal functional status, new signs/symptoms can appear after acute stroke hospitalisation in those patients with poorer functionality [20, 21].

In addition to its clinical impact, post-stroke OD and its main complications, malnutrition and pneumonia, have been associated with independent direct sanitary costs during acute hospitalisation and at long-term follow-up phases, direct non-sanitary costs and indirect costs associated with patient productivity losses [4]. Some health economic data on the cost and on sanitary resource consumption of post-stroke OD and its main complications have also been reported [5, 6, 22]. However, there is a lack of literature on the cost-effectiveness of the appropriate management of OD. A summary of the available literature in this field could help as a point of departure for future investigation aimed at studying the cost-effectiveness of these interventions to create an economic model of the disease. Considering that OD and its main complications, malnutrition and pneumonia, have been associated with high costs after stroke, the massive screening and specialised management of post-stroke patients with OD not only could significantly improve patients’ clinical outcomes and quality of life (QoL) but also significantly reduce costs. This systematic review will be the second part of a research project created to evaluate the specific burden of OD on health and social costs after stroke and the cost-effectiveness of appropriate management and treatment of this condition.

Management of post-stroke OD comprises the early and systematic evaluation of these patients’ deglutition (screening and assessment) and the management of the impairments in safety of swallow that can cause aspiration and aspiration pneumonia and the impaired efficacy of swallow that can lead to dehydration and malnutrition and subsequent impaired immunity and frailty. Finally, poor oral hygiene is prevalent among these patients and it is associated with oral colonisation by respiratory pathogens. Diagnosis of post-stroke OD includes the assessment of impaired biomechanics of swallowing function and the characterisation of dysfunctional sensorimotor integration processes involved in deglutition. The paradigm of treatment is changing from compensatory strategies to the promotion of brain plasticity aiming at the recovery of both impaired swallow and brain-related swallowing dysfunction [7, 23]. We believe that these two strong tendencies and the results of new randomised control trials will induce, in the near future, many changes in the management of post-stroke OD and future treatment of stroke will be very different from what it is today. With regard to management, there is evidence to show that increasing the levels of viscosity reduces the risk of airway penetration and aspiration and recent studies with gum-based thickeners show the specific range of viscosity values providing this therapeutic effect on safety of swallow [6]. Long-term studies showing the clinical impact of fluid thickening in post-stroke patients are clearly required. A minimal and massive intervention that has been proven to be useful and effective in older people with OD involves fluid and food texture adaptations, nutritional support and oral hygiene [24].

Innovative strategies that aim to restore the swallowing function have emerged during recent years. Those strategies include pharmacological and electrical peripheral (transcutaneous and intrapharyngeal stimulation) and central stimulation treatments such as tDCS and rTMS strategies [7]. Transcutaneous electrical stimulation (TES) is a safe and effective therapy for chronic post-stroke OD patients. A recent randomised study with a one-year follow up reported that the biomechanical effect of both sensory (SES) and motor (MES) electrical stimulation strategies improved the safety of swallow in post-stroke OD patients and reduced the need for fluid thickening without any major adverse event [25]. Moreover, recent data show that strategies aiming to neurostimulate the sensory pathway cause an immediate improvement in the excitability of the motor cortex (pharmacological modulation with capsaicin and intra-pharyngeal electrical stimulation) and of the pharyngeal sensory conduction (rTMS) [26]. These interventions are safe, simple, cost-effective and are based on scientific evidence collected over decades and will change the paradigm of treatment of post-stroke patients with OD [10].

## Data Availability

Not applicable.

## Abbreviations

CHEERS: Consolidated Health Economic Evaluation Reporting Standards
DALYs: Disability-adjusted life years
EMBASE: Excerpta Medica Database
ICER: Incremental cost-effectiveness ratio
ICUR: Incremental cost-utility ratio
MES: Motor electrical stimulation
MeSH: Medical Subject Heading
NIBS: Non-invasive brain stimulation techniques
NIHSS: National Institutes of Health Stroke Scale
OD: Oropharyngeal dysphagia
PEG: Percutaneous endoscopic gastrostomy
PRISMA: Preferred Reporting Items for Systematic Reviews and Meta-analysis
PRISMA-P: Preferred Reporting Items for Systematic Reviews and Meta-analysis-Protocols
PROSPERO: International Prospective Register of Systematic Reviews
QoL: Quality of life
QUALYs: Quality-adjusted life years
rTMS: Repetitive transcranial magnetic stimulation
SES: Sensory electrical stimulation
TES: Transcutaneous electrical stimulation
tDCS: Transcranial direct current stimulation
